# Population frequencies of thiopurine-related pharmacogenes in healthy individuals from Kosovo

**DOI:** 10.1038/s41439-026-00337-3

**Published:** 2026-02-27

**Authors:** Flaka Pasha, Dunja Urbančič, Gordana Gosheva, Bukurije Zhubi, Safete Maliqi Qormemeti, Shaip Krasniqi, Irena Mlinarič-Raščan

**Affiliations:** 1 Faculty of Pharmacy, University of Ljubljana, Ljubljana, Slovenia; 2 Department of Pharmacology with Toxicology, and Clinical Pharmacology, Faculty of Medicine, University of Prishtina, Hasan Prishtina, Kosovo; 3 National Center for Blood Transfusion in Kosovo, University Clinical Center of Kosovo, Prishtine, Kosovo

**Keywords:** Genetics research, Pharmacogenomics

## Abstract

Personalized thiopurine therapy is among the most established examples of pharmacogenomics translated into clinical practice. Variants in *TPMT* (rs1800462, rs1800460, rs1142345) and *NUDT15* (rs116855232) are recognized clinical predictors of thiopurine efficacy and toxicity. Additional variants in genes such as *PACSIN2* (rs2413739), *ITPA* (rs1127354) and *MTHFR* (rs1801133 and rs1801131), also contribute to variability in drug response. Here we characterize the frequency of the pharmacogenetic variants involved in thiopurine metabolism in a healthy population of Kosovo. We genotyped 299 healthy blood donors for polymorphisms. Among *TPMT* variant alleles, TPMT*3A was observed at a frequency of 2.0%, and the TPMT*3C at 0.1%. Notably, the *MTHFR* 677T variant (rs1801133) was significantly more frequent in the Kosovo population (49.8%) compared with the global and European frequencies. The minor allele frequency of *MTHFR* rs1801131 was 27.4%. Minor allele frequencies for *PACSIN2* rs2413739 and *ITPA* rs1127354 variants were 48.8% and 4.0%, respectively. Sequencing of *NUDT15* revealed six variants, with rs116855232 present at frequency of 0.8%. These findings provide important insights into the pharmacogenomic profile of the Kosovo population and support the implementation of pre-emptive genotyping to improve the safety and efficacy of thiopurine therapy in the region.

## Genetic variants impacting thiopurine response in Kosovo

Genetic inheritance affects how people respond to medications, which can lead to successful treatments or harmful side effects. This study focuses on thiopurines, drugs used for conditions such as leukemia and organ transplants. Researchers in Kosovo explored genetic differences that influence thiopurine effectiveness and safety. Researchers collected blood samples to analyze genetic variations in five key genes: *TPMT*, *NUDT15*, *MTHFR*, *ITPA* and *PACSIN2*. These genes are important because they affect how the body processes thiopurines. The study used techniques such as TaqMan genotyping and Sanger sequencing to identify genetic differences. The results showed that some genetic variations were more common in the Kosovo population compared with global averages. This information is crucial for developing personalized medicine strategies in Kosovo. The researchers concluded that understanding these genetic differences can help tailor treatments to reduce side effects and improve outcomes.

This summary was initially drafted using artificial intelligence, then revised and fact-checked by the author.

## Introduction

Genetic inheritance plays a crucial role in determining individuals’ response to therapy, influencing either favorable treatment outcomes or therapeutic failure accompanied by adverse effects^1^. Pharmacogenomics investigates how interindividual genetic variations impact patients’ response to therapy, aiming to enhance drug efficacy, while mitigating therapy-associated adverse effects^2^. In the field of pharmacogenomics and precision medicine, thiopurines have undergone comprehensive research and are now one of the best examples of its implementation into clinical practice^3^.

Thiopurines (6-mercaptopurine (6-MP), 6-thiogunanine (6-TG) and azathioprine (AZA)) are purine antimetabolites^4^ and immunosuppressant drugs^5^, frequently prescribed in the maintenance phase of acute lymphoblastic leukemia (ALL)^6^, for glucocorticoid-sparing remission maintenance of inflammatory bowel diseases^7^ and autoimmune disorders^8^, and in post-organ transplantation care^9^. Thiopurines are prodrugs that require metabolic activation to exert their clinical effects^10^. Mainly through incorporation into DNA and RNA^11^, active metabolites of thiopurines, 6-thioguanine nucleotides (6-TGNs), induce S-phase arrest and trigger programmed cell death^12^. The efficacy and safety of thiopurine treatment is influenced by thiopurine *S*-methyltransferase (TPMT), an enzyme that deactivates thiopurines by *S*-methylation toward the inactive metabolite 6-methylmercaptopurine (6-MMP), utilizing *S*-adenosyl methionine (SAM) as a methyl donor^13,14^. Four genetic variants in the gene encoding TPMT, namely TPMT *2, *3A, *3B and *3C, have been recognized to contribute to reduced TPMT enzymatic activity in individuals^15^. Patients who are wild-type homozygotes (TPMT*1/*1) exhibit normal TPMT enzyme activity, while heterozygotes and variant homozygote individuals for TPMT*2, *3A, *3B and *3C alleles show intermediate and low enzyme activity, respectively^16^. Decreased enzymatic activity of TPMT leads to higher concentration of 6-TGNs, greater cytotoxicity and higher risks of life-threatening adverse effects such as myelotoxicity^17^, secondary neoplasms^18^ and superimposed infections^19^.

**Fig. 1:**
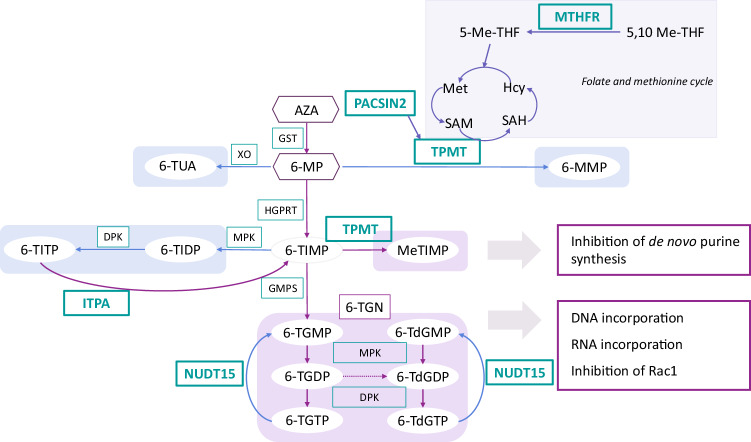
Overview of key enzymes contributing to interindividual variability in the thiopurine response. 5-Me-THF, 5-methyltetrahydrofolate; 5,10 Me-THF, 5,10-methylenetetrahydrofolate; 6-MeTIMP (6-methyl thioinosine monophosphate), 6-MMP, 6-methylmercaptopurine; 6-MP, 6-mercaptopurine; 6-TdGDP, 6-thio-deoxyguanosine diphosphate; 6-TdGMP, 6-thio-deoxyguanosine monophosphate; 6-TdGTP, 6-thio-deoxyguanosine triphosphate); 6-TG, 6-thioguanine; 6-TGDP, 6-thioguanosine diphosphate; 6-TGMP, 6-thioguanosine monophosphate; 6-TGN, 6-thioguanine nucleotide; 6-TGTP, 6-thioguanosine triphosphate; 6-TIDP, 6-thioinosine diphosphate; 6-TIMP, 6-thioinosine monophosphate; 6-TITP, 6-thioinosine triphosphate; 6-TUA, 6-thiouric acid; AZA, azathioprine; DNA, deoxyribonucleic acid; DPK, diphosphokinase; GMPS, guanosine monophosphate synthetase; GST, glutathione *S*-transferase; HGPRT, hypoxanthine-guanine phosphoribosyl transferase; Hcy, homocysteine; ITPA, inosine triphosphate pyrophosphatase; Met, methionine; MPK, monophosphokinase; MTHFR, methylenetetrahydrofolate reductase; NUDT15, nudix hydrolase 15; PACSIN2, protein kinase C and casein kinase substrate in neurons 2; RNA, ribonucleic acid; SAH, *S*-adenosylhomocysteine; SAM, *S*-adenosyl methionine; TPMT, thiopurine *S*-methyl transferase; XO, xanthine oxidase.

Diverse thiopurine response among individuals cannot solely be attributed to *TPMT* polymorphisms (Fig. 1). Another important pharmacogene in thiopurine treatment already implemented into clinical practice is nudix hydrolase 15 (*NUDT15*)^20^. Because of its efficient hydrolysis of deoxyguanosine triphosphate (dGTP), NUDT15 is a crucial enzyme in purine nucleotide homeostasis. As active metabolites of thiopurines are analogs of dGTP, genetic polymorphisms in *NUDT15* that decrease enzyme activity (NUDT15*3, *2, *9 and *14) cause reduced 6-TGN levels and decreased thiopurine toxicity^21^. In addition, the genetic polymorphism rs2413739 (T > C) in *PACSIN2* also influences TPMT activity. In pediatric patients, wild-type genotype was associated with lower TPMT activity and increased patients’ risk of gastrointestinal and hematologic toxicity^22^. Furthermore, inosine triphosphate pyrophosphatase (ITPA) catalyzes the pyrophosphohydrolysis of inosine triphosphate (ITP) to inosine monophosphate (IMP), preventing the accumulation of ITP and 6-thioxanthosine triphosphate into cells, rescuing them from apoptosis and reducing the risk of myelotoxicity and hepatotoxicity in patients treated with thiopurines. Studies have shown that the presence of at least one nonfunctional *ITPA* allele (either the 94C>A or IVS2+21A>C variant) is linked to a longer event-free survival compared with patients with the wild-type *ITPA* genotype. Moreover, patients with at least one nonfunctional *ITPA* allele had a reduced risk of experiencing early relapse or bone marrow relapse^23^. Lastly, single-nucleotide polymorphisms (SNPs) in *MTHFR* (rs1801133 and rs1801131) lead to reduced enzyme activity and have been recognized to increase 6-MP toxicity in patients with ALL. MTHFR catalyzes the formation of 5-methylenetetrahydrofolate, facilitating the conversion of homocysteine to methionine and thereby restoring the methylation potential of the cell. By reducing the availability of methionine and consequently SAM, genetic polymorphisms in *MTHFR* may decrease the methylation of thiopurines^14,24^.

Genotyping for common and clinically relevant genetic polymorphisms related to thiopurine metabolism and folate cycle before application of thiopurines can thus mitigate adverse thiopurine effects, without compromising the disease relapse rate^25^. Guidelines from the Clinical Pharmacogenetics Implementation Consortium (CPIC), the Dutch Pharmacogenetics Working Group, and the Canadian Pharmacogenomics Network for Drug Safety recommend pre-emptive genotyping for variant alleles when there is a well-established functional impact; a global minor allele frequency (MAF) >1% or a MAF >1% in a specific population; availability of reference materials; feasibility of testing using standard laboratory methods; or a well-documented association between the variant allele and drug response^26^. According to CPIC and Dutch Pharmacogenetics Working Group guidelines, only genetic polymorphisms in *TPMT* and *NUDT15* are currently recommended to be assessed before treatment^15^. Based on genotype, normal metabolizers for TPMT and NUDT15 should start with standard thiopurine doses, while intermediate metabolizers should receive 30–80% or 50–80% of standard AZA/6-MP or 6-TG dose, respectively. Poor metabolizers diagnosed with malignancies should initiate 6-MP at doses reduced tenfold and administered three times weekly, whereas in nonmalignant conditions, alternative treatments to AZA should be considered^27^.

Despite the well-documented association between genetic markers and thiopurine metabolism, their integration into clinical practice poses notable challenges^28^, including the absence of pre-emptive genotyping services in certain healthcare systems. Therefore, we aim to set the basis of genetic testing for individuals undergoing thiopurine therapy in Kosovo, by developing a framework for pharmacogenetic testing for *TPMT*, *NUDT15*, *MTHFR*, *ITPA* and *PACSIN2* polymorphisms. As one of the requirements for implementing genetic polymorphism testing into clinical practice is a MAF greater than 1% in the general or a specific population, this study explores the frequency of genetic polymorphisms previously reported as (potential) biomarkers for thiopurine treatment in a healthy population from Kosovo. This study provides valuable insights into the genetic landscape within the Kosovo population and highlights both similarities and unique characteristics that contribute to enriching the map of human genetic variation worldwide.

## Materials and methods

### Study population

This study includes 299 healthy blood donors, randomly selected from the National Center for Blood Transfusion at the University Clinical Center of Kosovo. Inclusion criteria comprised age between 18 and 65 years, weight over 50 kg, hemoglobin levels within the reference range, normal blood pressure, no high-risk behaviors and no prior travel to areas endemic for malaria or Zika virus. The study was conducted in accordance with the Declaration of Helsinki, for studies involving humans. The study was approved by the Ethics Committee of the Faculty of Medicine, University of Prishtina ‘Hasan Prishtina’ (approval no. 4095, dated 31 May 2019) and by the University Clinical Center of Kosovo (approval no. 1429, dated 3 June 2019). Informed written consent, including the demographic questionnaire, was obtained from all participants involved in the study. From each study participant, 3–5 ml of whole venous blood was collected.

### DNA isolation and genotyping

DNA was isolated from 200 µl of whole blood from each participant using the spin protocol for DNA purification, following the manufacturer’s instructions (QIAGEN QIAamp DNA Mini Kit).

Genetic polymorphisms in *TPMT*, *PACSIN2*, *ITPA* and *MTHFR* were determined by TaqMan SNP genotyping assay (Thermo Fisher Scientific, Applied Biosystems), following the manufacturers’ instructions. For quantitative PCR, we used the LightCycler 480 system (Roche). SNPs and part numbers of all TaqMan SNP genotyping assays are listed in Table 1.

**Table 1:** The list of analyzed SNPs, their rs numbers, nucleotide changes and TaqMan Assay IDs.

rs number	Gene symbol	Nucleotide change	TaqMan assay ID
rs1800462	*TPMT*	c.238G>C (*2)	C_12091552_30
rs1800460	*TPMT*	c.460G>A (*3B)	C_30634116_20
rs1142345	*TPMT*	c.719A>G (*3C)	C_19567_20
rs2413739	*PACSIN2*	c.IVS2+21A>G	C___2503304_20
rs1127354	*ITPA*	c.94C>A	C__27465000_10
rs1801133	*MTHFR*	c.677C>T	C___1202883_20
rs1801131	*MTHFR*	c.1298A>C	C____850486_20

rs number, reference SNP cluster ID.

Genetic polymorphisms in *NUDT15* were determined by Sanger sequencing of exon 1 and exon 3, as the most relevant *NUDT15* genetic variants lie within these regions. The procedure followed essentially the same protocol as previously reported^29^, with primers listed in Table 2. Samples were sequenced by Microsynth AG.

**Table 2:** Primer pairs used for sequencing of exon 1 and exon 3 of *NUDT15*.

Primer	Sequence (5′–3′)
*NUDT15* exon1 forward	CAAAGCACAACTGTAAGCGAC
*NUDT15* exon1 reverse	CACACCTCACAGACGAACTC
*NUDT15* exon3 forward	CAAGCAAATGCAAAGCATCAC
*NUDT15* exon3 reverse	GGCTGAAAGAGTGGGGGATA

### Statistical analysis

Sequences were determined using Finch TV software (Geospiza)^30^. Distributions of genotypes and possible deviations from the Hardy–Weinberg equilibrium were assessed by Fisher’s exact test. Differences between Kosovo, global and European allelic frequencies of SNPs were analyzed using Fisher’s exact test or chi-square test. The threshold for significance was set at *P* < 0.05.

## Results

### Population characteristics

To assess the distribution of genetic polymorphisms related to thiopurine treatment in the population of Kosovo, we collected blood samples from 299 consenting healthy blood donors. The mean age of the healthy blood donor cohort was 37 years. Among the 299 participants, 84% (*n* = 252) were male and 16% (*n* = 47) were female. The vast majority, 99% (*n* = 297), identified as Kosovars by nationality, while two participants identified as Turk (*n* = 1, 0.003%) and Egyptian (*n* = 1, 0.003%) minorities. Other ethnic groups were not represented in this study cohort. The donors were recruited from all regions of Kosovo, with 41% from Prishtina, 8% from Podujeva, 5% each from Gjilan, Peja, and Prizren, and the remaining participants from other cities, each contributing less than 5%.

Regarding blood types, group 0 was the most prevalent, accounting for 44% (*n* = 130) of the cohort, followed by type A at 39% (*n* = 118), type B at 12% (*n* = 37) and group AB at 5% (*n* = 14).

### Frequencies of genetic variants in *TPMT*, *ITPA*, *PACSIN2*, *MTHFR* and *NUDT15* in Kosovo population

To determine the frequency of the most relevant genetic polymorphisms in the genes implicated in thiopurine metabolism and folate cycle, we used TaqMan genotyping for variants in *TPMT* (rs1800462, rs1800460 and rs1142345), *ITPA* (rs1127354), *PACSIN2* (rs2413739) and *MTHFR* (rs1801133 and rs1801131) and Sanger sequencing for *NUDT15*. All 299 individuals in our study were genotyped for SNPs in *TPMT*, *MTHFR*, *PACSIN2* and *ITPA*. A subpopulation of donors (*N* = 184) was tested for *NUDT15*. In Table 3, we present the frequencies of alleles and genotypes for each gene in detail.

**Table 3:** Allele and genotype frequencies of polymorphisms influencing thiopurine metabolism and folate cycle among 299 healthy blood donors from Kosovo.

Gene	Allele and SNP ID	Nucleotide change	Major > minor allele	*N*^a^	Major allele homozygote (*N*)	Heterozygote (*N*)	Minor allele homozygote (*N*)	MAF	HWE (*P* value)
*TPMT*	TPMT*2, rs1800462	NC_000006.11:g.18143955C>G	C > G	277	277	0	0	0	>0.9999
	TPMT*3A, rs1800460, rs1142345	NC_000006.11:g.18139228C>T NC_000006.11:g.18130918T>C	C > T T > C	299	287	12	0	0.02	>0.9999
	TPMT*3C, rs1142345	NC_000006.11:g.18130918T>C	T > C	299	286	13	0	0.001	>0.9999
*MTHFR*	rs1801133	NC_000001.10:g.11856378G>A	G > A	299	80	140	79	0.498	0.748
	rs1801131	NC_000001.10:g.11854476T>G	T > G	299	156	122	21	0.274	0.9663
*PACSIN2*	rs2413739	NC_000022.10:g.43397036C>T	C > T	297	84	136	77	0.488	0.6116
*ITPA*	rs1127354	NC_000020.10:g.3193842C>A	C > A	299	275	24	0	0.04	>0.9999
*NUDT15*	rs45465203	NC_000013.10:g.48612214G˃A	G ˃ A	184	137	45	2	0.133	0.8781
	rs61973267	NC_000013.10:g.48619942G˃A	G ˃ A	184	156	26	2	0.082	0.912
	rs79687000	NC_000013.10:g.48612157C˃T	C ˃ T	184	179	5	0	0.021	>0.9999
	rs377238223	NC_000013.10:g.48612050C˃G	C ˃ G	184	183	1	0	0.003	>0.9999
	rs746071566	NC_000013.10:g.48611920GAGTCG[2]	delGAGTCG	184	183	1	0	0.003	>0.9999
	NUDT15*3, rs116855232	NC_000013.11:g.48045718C>T	C > T	184	182	1	1	0.008	0.6229

The threshold for significance was set to *p* < 0.05.

^a^The number of the individuals successfully assessed.

*HWE* Hardy–Weinberg equilibrium, *MAF* minor allele frequency.

No significant deviation from Hardy–Weinberg equilibrium was observed for any of the investigated genetic polymorphisms (*P* = 0.6116-0.999; Fisher’s exact test). In the Kosovo population, we observed the following MAFs for alleles in the *TPMT*, *PACSIN2*, *ITPA* and *MTHFR* genes. The TPMT*2 allele was not detected (MAF of 0%). The TPMT*3A allele, a diplotype of TPMT*3B and TPMT*3C, was found in 12 heterozygous individuals with a frequency of 2.0%. The TPMT*3C allele alone was identified in one heterozygous individual, at a frequency of 0.1%. For the *PACSIN2* rs2413739 variant, the MAF was 48.8%, while for the *ITPA* rs1127354 variant the MAF was 4%. For the *MTHFR* gene, the rs1801133 variant had a MAF of 49.8%, and the rs1801131 variant was observed at a frequency of 27.4%.

We identified six variants in *NUDT15* by Sanger sequencing of exon 1 and exon 3. The identified variants were *NUDT15* rs45465203 with a MAF of 13.3%, followed by rs61973267 (8.2%), rs79687000 (2.1%), rs116855232 (0.8%), rs377238223 (0.4%) and rs746071566 (0.4%).

### Comparison of Kosovo population with general global and European populations

To assess similarities and differences in the MAFs of polymorphisms associated with thiopurine metabolism, we compared data from the Kosovo population with global and European reference populations using the gnomAD database and applied the Fisher’s exact test or chi-square test, as appropriate^31^. We present our findings in Table 4.

**Table 4:** Comparison of minor allele frequencies (MAFs) (%) for common polymorphisms related to thiopurine metabolism, between global, European and Kosovo population.

Pharmacogene	*TPMT*^b^			*NUDT15*						*ITPA*	*PACSIN2*	*MTHFR*	
Level of evidence (CPIC/PharmGKB)^a^	**A/1A**	**A/1A**	**A/1A**	**NA**	**NA**	**NA**	**A/1A**	**NA**	**A/1A**	**NA/3**	**NA/3**	**NA/4**	**NA**
MAF (%)	rs1800462	rs1800460	rs1142345	rs45465203	rs61973267	rs79687000	rs746071566	rs377238223	rs116855232	rs1127354	rs2413739	rs1801133	rs1801131
Kosovo population	0.0	2.0	2.1	13.3	8.2	2.7	0.27	0.27	0.80	4.0	48.8	49.8	27.4
Global population	0.21	3.5	4.3	11.9	6.4	2.8	0.59	0.0047	1.2	7.5	42.6	31.8	30.3
*P* value for Kosovo versus global population	0.6373	0.0595	**0.0145**	0.4210	0.2331	0.9928	0.7301	**0.0175**	0.8059	**0.0016**	**0.0027**	**<0.0001**	0.1343
European population	0.25	4.2	4.6	13.1	6.8	2.2	0.28	0.0055	0.25	7.3	44.0	33.7	31.3
P value for Kosovo versus European population	0.652	**0.0113**	**0.0051**	0.8773	0.1709	0.4817	>0.9999	**0.0205**	0.0692	**0.0022**	**0.0088**	**<0.0001**	**0.0446**

The threshold for significance (in bold) was set to *P* < 0.05.

^a^Level of evidence for thiopurines were retrieved from CPIC guidelines and ClinPGx database^32^. CPIC levels (gene–drug evidence): A, strong actionable; B, moderate; C/D, limited. ClinPGx (that is, PharmGKB) levels (variant–drug evidence)^33^: 1A, guideline-supported/actionable; 1B–2B, moderate; 3–4, preliminary. NA, no level of evidence or clinical guideline available.

^b^TPMT*2 corresponds to rs1800462; TPMT*3A to the haplotype of rs1800460 and rs1142345; and TPMT*3C to rs1142345.

We observed significant differences between Kosovo and global/European populations, especially in frequencies of *MTHFR* polymorphisms; however, the MAFs of certain other polymorphisms in the Kosovo population, particularly those of *ITPA*, *PACSIN2* and *TPMT*, also showed significant deviations from global and/or European populations.

The global and European MAF for the *TPMT* rs1800462 variant is 0.2%, with no statistically significant differences compared with the Kosovo population, in which no carriers of this variant were detected. Similarly, there were no significant differences in the MAF of the *TPMT* rs1800460 variant between Kosovo (MAF of 2%) and global (3.5%), while there was a significant difference (*P* = 0.0113) with the European population (4.2%). By contrast, for the *TPMT* rs1142345 variant, the global population MAF is 4.3% and the European population MAF is 4.6%, whereas the Kosovo population MAF was slightly lower at 2.1%, representing a statistically significant difference (*P* = 0.0145 and 0.0051, respectively).

Furthermore, the MAFs for the *NUDT15* rs45465203, rs61973267, rs79687000, rs116855232 and rs746071566 variants in the Kosovo population were not statistically different from those in the European population. The *NUDT15* variant rs377238223 showed a statistically significant deviation in allele frequency in the Kosovo population; however, this finding was based on a single individual and may represent a chance occurrence.

We also examined the MAF for the *ITPA* rs1127354 variant in the Kosovo population, which was 4%. This was significantly lower than the MAF of this variant in the global (7.5%) and the European (7.3%) population, with *P* values of <0.0016 and <0.0022, respectively. In addition, the MAF for the *PACSIN2* rs2413739 variant in the Kosovo population was significantly higher compared with the global population (*P* < 0.0027) and European population (*P* < 0.0088).

When comparing MAFs of *MTHFR* rs1801131, no statistically significant differences were observed between the Kosovo population (27.4%) and the global population (30.3%). However, a slight but statistically significant difference was found when compared with the European population (*P* = 0.0446). By contrast, the MAF of the *MTHFR* rs1801133 variant (677C>T) in the Kosovo population was 49.8%, which is significantly higher (*P* < 0.0001) than the MAF in both the global population (31.8%) and the European population (33.7%). Notably, this frequency is comparable to that reported for the Italian population (46.7%), with no statistically significant difference observed.

## Discussion

This study presents a comprehensive analysis of genetic polymorphisms associated with thiopurine metabolism and the folate pathway in a healthy population of Kosovo, evaluating the allele frequencies of pharmacogenes relevant to thiopurine therapy in this population. The findings provide essential information to guide healthcare practitioners and policymakers in establishing and optimizing pre-emptive genotyping services in Kosovo.

We genotyped 299 healthy blood donors from the National Center for Blood Transfusion of Kosovo and assessed the cohort for 7 genetic polymorphisms in 4 different genes, that is, *TPMT*, *ITPA*, *PACSIN2* and *MTHFR*, and additionally identified six genetic variants in *NUDT15*. These specific variants were selected on the basis of established evidence of their association with thiopurine efficacy and toxicity^26^. Among the investigated variants, *TPMT* and *NUDT15* polymorphisms are supported by high-level evidence for clinical implementation in thiopurine pharmacogenomics (Table 4). By contrast, the evidence for *PACSIN2*, *ITPA* and *MTHFR* variants is limited to individual clinical reports suggesting possible effects on thiopurine dosing or toxicity, while the additional *NUDT15* variants were identified by sequencing in this study and have no previous association with thiopurine treatment.

The most clinically relevant *TPMT* variants, TPMT*3C and TPMT*3A, were found at low frequencies in the Kosovo population. Although the TPMT*3A allele is the most common TPMT-activity-affecting variant in the European population, the Kosovo population had a MAF of 2.0%, which is lower than that reported in other European cohorts (2.7–4.5%)^32,33^. The TPMT*3C allele, which is more prevalent in East Asian and African populations, was rare (0.1%), aligning with findings from other Eastern European countries such as Poland (0.1%), Sweden (0.2%) and Bulgaria (0.2%)^34–36^.

In contrast to the TPMT*3A allele, *NUDT15* polymorphisms showed frequencies consistent with reports from non-Finnish European populations and global population. Two individuals carried the clinically important NUDT15*3 variant (rs116855232), and one carried rs746071566, a microsatellite insertion previously reported not to be associated with thiopurine-induced myelosuppression^37^. The Kosovo MAF for rs61973267 was similar to that of other European populations, including Finnish (6.1%), British (8.8%) and Spanish (5.1%) cohorts^38^.

The MAF of the *ITPA* rs1127354 variant, associated with thiopurine-related adverse effects^39^, was significantly lower in Kosovo population than in global and broader European datasets. However, the MAF remained above 1%, supporting the consideration of this variant in pharmacogenomic screening. Although current evidence is insufficient to support the routine inclusion of *PACSIN2* rs2413739 in pre-emptive testing guidelines, the slightly higher MAF observed in the Kosovo population highlights its potential relevance and supports further investigation into its clinical utility in thiopurine pharmacogenomics^40^.

A particularly interesting result was the high frequency of *MTHFR* rs1801133 (c.677C>T; p.Ala222Val) variant, with a MAF of 49.8%, which is significantly higher than the MAF in global (31.8%) and European populations (33.7%), including Finnish (27.3%)^41^ and British (32.4%) subpopulations^42^. Higher *MTHFR* C677T allele frequencies have been previously reported in Hispanic/Latin American populations (CLM, MXL, PEL and PUR), in certain regions of China (CHB) and in specific parts of Italy (TSI)^43,44^. The comparison analysis across populations with relatively high MAFs (>45%) for *MTHFR* rs1801133 showed that the Kosovo population exhibits a similar frequency of the *MTHFR* 677T allele (Supplementary Table 1). When extended to all polymorphisms investigated, correlation analysis indicated that the Kosovo population is most similar to other European populations, particularly those from Southern Europe (for example, Toscani Italians), while also sharing allelic trends with Latin American groups and the Finnish population (Supplementary Table 1). The *MTHFR* 677 variant leads to a thermolabile MTHFR enzyme with considerably reduced MTHFR activity—about 60% in heterozygous and as low as 10% in variant homozygous individuals^45–47^. Reduced MTHFR activity disrupts the efficiency of one-carbon metabolism, which is crucial for cell differentiation and growth^48^. This genetic polymorphism is thus strongly associated with elevated homocysteine levels, as well as increased susceptibility to congenital birth defects, such as neural tube defects^49^ and congenital heart defects^50^. Given the high MAF of the *MTHFR* rs1801133 variant observed in the Kosovo population, not only could there be an increased risk of impaired thiopurine methylation, but public health strategies should also consider targeted folate supplementation programs—particularly for women of reproductive age—to mitigate the risk of folate-related birth defects^51^.

Some limitations of our study should be acknowledged. The results are obtained from a specific healthy blood donor sample cohort and may not fully represent all subpopulations in Kosovo, especially those with diverse ethnic backgrounds. Given that the frequency of SNPs can differ across ethnic groups, future studies should include multiple ethnic groups to validate the broader applicability of our results. In addition, our cohort consisted predominantly of males (84%), which may not fully capture gender differences in the population, despite the absence of X-linked SNPs. Therefore, future studies with more ethnically diverse populations, incorporating a wider array of genetic and nongenetic factors, would not only improve the robustness of allele frequency estimates but also enhance the clinical utility of pharmacogenomic recommendations.

## Conclusion

This study represents an important first step in characterizing the pharmacogenetic variation related to thiopurine metabolism in a healthy population of Kosovo. By providing allele frequency data for key variants in *TPMT*, *NUDT15*, *ITPA*, *PACSIN2* and *MTHFR*, these findings offer essential insights into the genetic landscape of Kosovo population. They reveal important implications for the development of personalized thiopurine treatment strategies. Although advancements in pharmacogenomics and precision medicine are evident globally, successful integration of pre-emptive approaches into the healthcare system in Kosovo will require overcoming barriers such as limited awareness, a lack of trained personnel, costs and accessibility of services, and the need for infrastructure to support continuous patient monitoring. The study lays the groundwork for future efforts for the implementation of pharmacogenomic data into clinical decision-making in Kosovo, aiming to enhance patient safety and treatment efficacy.

## Supplementary information

Supplementary Table 1

## Data Availability

The data analyzed in this study are available from the corresponding author upon request.
